# Effect of Hyperglycemia-Related Acute Metabolic Disturbance on Thyroid Function Parameters in Adults

**DOI:** 10.3389/fendo.2022.869869

**Published:** 2022-05-12

**Authors:** Yuichiro Iwamoto, Tomohiko Kimura, Fuminori Tatsumi, Toshitomo Sugisaki, Masato Kubo, Erina Nakao, Kazunori Dan, Ryo Wamata, Hideyuki Iwamoto, Kaio Takahashi, Junpei Sanada, Yoshiro Fushimi, Yukino Katakura, Masashi Shimoda, Shuhei Nakanishi, Tomoatsu Mune, Kohei Kaku, Hideaki Kaneto

**Affiliations:** Department of Diabetes, Endocrinology and Metabolism, Kawasaki Medical School, Kurashiki, Japan

**Keywords:** diabetic ketosis, diabetic ketoacidosis, hyperglycemic hyperosmolarity syndrome, non-thyroidal illness, thyroid function

## Abstract

Non-thyroidal illness (NTI) is a condition in which the hypothalamic-pituitary-thyroid system and thyroid hormone metabolism are abnormal due to non-thyroidal diseases. Although NTI has been reported to occur in hyperglycemic emergencies in children, there have been few studies in adult cases. In this study, we examined adult patients with hyperglycemia regarding the frequency of NTI and its triggers. Adult diabetic patients who were hospitalized for diabetic ketosis (DK), diabetic ketoacidosis (DKA), or hyperglycemic hyperosmolarity syndrome (HHS) were included in the study. Compared with the DK group, the DKA and HHS groups had higher admission blood glucose, Anion Gap, serum osmolality, creatinine, and urea nitrogen, and lower pH and eGFR. The frequency of NTI in the DKA, HHS, and DK groups was 80%, 70%, and 50%, respectively, and thyroid stimulating hormone (TSH) and free thyroxine 3 (FT3) were significantly improved after treatment for hyperglycemia. Multiple regression analysis showed a significant correlation between the decrease in FT3 level and 3-hydroxybutyrate and albumin. Acute metabolic failure associated with hyperglycemia tends to be associated with a high rate of NTI and low FT3 levels at the start of treatment. The data in this study clearly shows that transient NTI is frequently observed in subjects with acute metabolic disorders such as DKA, HHS and DK. In addition, we should bear in mind that thyroid hormone replacement therapy is not necessary in subjects with NTI due to DKA, HHS and DK, especially when overt symptoms of hypothyroidism are not observed.

## Introduction

Non-thyroidal illness (NTI) is a condition in which the hypothalamic-pituitary-thyroid system and thyroid hormone metabolism are abnormal due to non-thyroidal diseases, and it is an adaptive response to reduce catabolism for biological defense in severe diseases (1). In non-thyroidal diseases, low T3 syndrome is the most common type, and severe infections such as sepsis can lead to insufficient secretion of thyrotropin-releasing hormone (TRH) and thyroid-stimulating hormone (TSH) due to inflammatory cytokines (2, 3). The mechanism of thyroid hormone depletion in NTI is that corticosteroids, inflammatory cytokines, and other factors reduce the 5’-deiodination response and impair the conversion of T4 to T3. It has been suggested that decreased TRH secretion due to decreased leptin and decreased TSH secretion due to inflammatory cytokines are involved in hypothyroidism in subjects with NTI (4).

Diabetes mellitus is a disease characterized by hyperglycemia due to inadequate insulin secretion and/or sensitivity. Diabetes cases with HbA1c >12% show a decrease in serum T3 concentration and a decrease or normal range of TSH (5). There are reports showing that serum T3 concentration is correlated with the management of diabetes (6). Factors such as dehydration, fasting, and excessive consumption of soft drinks can lead to metabolic disorders such as diabetic ketoacidosis (DKA), hyperglycemic hyperosmolar syndrome (HHS), and diabetic ketosis (DK). A study reported by Shao et al. showed that pediatric patients with DKA and DK had abnormal thyroid function and that serum FT3 levels were correlated with β-hydroxybutyrate and bicarbonate ions (7). Although it has been reported that DKA and DK are involved in the pathogenesis of NTI in pediatric cases, there has been only a few case reports of HHS complicated by NTI or systemic inflammatory response syndrome (SIRS) in adult cases. Although there have been case reports of HHS complicated by NTI (8), no study has examined the impact of hyperglycemia-related metabolic dysfunction on thyroid dysfunction in adults. In the present study, we investigated the effects of DKA, HHS and DK on thyroid function.

## Materials and Methods

### Study Subject

We performed this study with hospitalized patients retrospectively in our institution from April 1st in 2016 to Jone 30th in 2021 at the Department of Diabetes, Metabolism and Endocrinology, Kawasaki Medical School, Kurashiki, Japan. The study protocol including the Opt-out informed consent was approved by Institutional Review Board of Kawasaki Medical School (No. 5375-00). And the study was conducted in accordance with the Declaration of Helsinki. Since this study was retrospective, instead of obtaining informed consent from each patient, we provided public information about the study *via* the hospital homepage.

The criteria for diagnosing DKA, HHS, and DK were based on the criteria posted by the Japanese Diabetes Association. DKA was defined as pH less than 7.30, AnionGap greater than 12.0, and increased ketone bodies; HHS was defined as blood glucose level greater than 600 mg/dL and serum osmolality greater than 320 mOsm/kg; DK was defined as a diagnosis of diabetes mellitus and a state of ketosis that did not lead to acidosis. The definition of NTI in this study was as follows: thyroid hormone levels deviated from institutional reference values and were not accompanied by an elevated TSH. In addition, only cases in which other thyroid diseases could be excluded by measurement of thyroid-related autoantibodies and thyroid ultrasonography were included in the analysis.

We initially selected 56 patients who were ≥ 20 years old and were hospitalized in our institution for management of DKA, HHS or DK and whose thyroid hormones were measured. All patients admitted for the management of DKA, HHS or DK were included in the study. Patients with autoimmune thyroid disease, using thyroid medication or corticosteroids were also excluded from the analysis (4 patients). Finally, 52 patients were included: 20 patients in the DKA group, 10 patients in the HHS group, and 22 patients in the DK group.

### Methods

On admission, diabetes- and thyroid-related parameters, arterial blood gas, liver function, renal function, blood pressure, body weight and body mass index (BMI) were assessed. During hospitalization, all patients were treated with intensive insulin therapy. Thyroid-associated antibodies (TRAb, TSAb, anti-TPO and anti-Tg antibodies) were measured and thyroid ultrasonography was performed in patients with abnormal thyroid hormone levels on admission. Thyroid hormone levels were retested when ketosis was improved to normal range or at discharge.

### Statistical Analysis

The clinical characteristics of the subjects used in the analysis were age, laboratory findings on admission, and thyroid-related parameters at discharge. Group differences in each index among the DKA, HHS and DK groups were analyzed using the Tukey Kramer method and unpaired t tests. Changes in thyroid-related parameters before and after treatment were analyzed using the corresponding t-test. Multivariate linear regression analysis was used to test for factors contributing to thyroid-related parameters. Statistical software was Excel Statistics for Mac version 16.54 (Social Research Information Co., Ltd., Tokyo) and JMP version 16.0.1 (SAS Institute Inc.).

## Results

### Clinical Characteristics

The clinical characteristics of the patients in this study are shown in **Table 1**. HbA1c (National Glycohemoglobin Standardization Program [NGSP]), 12.1 ± 3.8%, 12.4 ± 3.1% and 12.7 ± 2.5%; glycoalbumin: 42.5 ± 11.4%, 41.8 ± 11.8% and 36.6 ± 12.6% in DKA, HHS and DK group, respectively, suggesting the presence of marked hyperglycemia in all groups. There was no difference in HbA1c and glycoalbumin levels among the three groups. Total ketone bodies were 4660.1 ± 4027.3 μmol/L in DK group and 10115.4 ± 5157.3 μmol/L in DKA group (p<0.005); total ketone bodies in HHS were 4660.1 ± 4027.3 μmol/L, not different from DK. In the evaluation of thyroid hormone levels, FT3 was predominantly lower in DKA group, 2.17 ± 0.73 pg/mL compared to 2.76 ± 0.40 pg/mL in DK group (p<0.05). Thyroid hormone levels were not different between HHS and DK. pH and eGFR were lower in DKA and HHS groups compared to DK group, and Anion Gap, serum osmolality, creatinine, and urea nitrogen were higher in DKA and HHS groups.

**Table 1:** Comparison of various values among DK, DKA and HHS group on admission.

Parameter	DK group (n = 22)	DKA group (n=20)	p value (vs DK group)	HHS group (n=10)	p value (vs DK group)
Male/female	13/9	10/10		7/3	
Age (years)	50.3 ± 19.2	58.4 ± 20.2	n.s	70.0 ± 15.5	<0.05
Duration of diabetes (years)	8.0 ± 8.3	12.7 ± 11.7	n.s	9.4 ± 13.3	n.s
Body weight (kg)	69.9 ± 23.3	56.0 ± 15.0	<0.05	61.8 ± 22.0	n.s
BMI (kg/m^2^)	25.2 ± 6.4	22.0 ± 4.9	n.s	23.2 ± 6.6	n.s
Diabetes type 1/2	5/17	3/17		0/10	n.s
Systolic blood pressure (mmHg)	135.9 ± 27.4	140.3 ± 29.8	n.s	135.1 ± 14.7	n.s
Diastolic blood pressure (mmHg)	87.5 ± 16.7	79.6 ± 25.6	n.s	84.9 ± 9.1	n.s
Pulse rate (beats per minute)	96.2 ± 14.1	107.2 ± 19.9	n.s	101.4 ± 27.4	n.s
Body temperature (°C)	37.0 ± 0.6	36.2 ± 1.6	n.s	36.3 ± 0.9	<0.05
Blood glucose levels (mg/dL)	443.0 ± 161.5	645.7 ± 260.4	<0.05	751.8 ± 146.9	<0.0005
HbA1c (%, NGSP)	12.7 ± 2.5	12.1 ± 3.8	n.s	12.4 ± 3.1	n.s
Glycoalbumin (%)	36.6 ± 12.6	42.5 ± 11.4	n.s	41.8 ± 11.8	n.s
Urine ketone qualitative	2.2 ± 0.9	2.0 ± 1.0	n.s	0.8 ± 0.9	<0.005
Total ketone body (μmol/L)	4027.3 ± 3342.9	10115.4 ± 5157.3	<0.0005	4660.1 ± 4027.3	n.s
Acetoacetic acid (μmol/L)	1237.7 ± 1074.1	3145.4 ± 1717.5	<0.0005	1166.7 ± 1365.1	n.s
3-Hydroxybutyric acid (μmol/L)	2789.6 ± 2382.6	6965.5 ± 3594.2	<0.0005	42457.7 ± 3226.1	n.s
pH	7.40 ± 0.03	7.19 ± 0.12	<0.0005	7.36 ± 0.04	<0.05
Anion Gap	16.10 ± 4.12	23.05 ± 6.20	<0.0005	21.40 ± 6.64	<0.05
Serum osmotic pressure (mOsm/kg)	294.1 ± 17.0	335.2 ± 38.2	<0.0005	337.9 ± 18.7	<0.0005
TSH (μU/mL)	1.07 ± 0.58	1.07 ± 1.26	n.s	1.27 ± 0.95	n.s
FT3 (pg/mL)	2.76 ± 0.40	2.17 ± 0.73	<0.05	2.51 ± 0.51	n.s
FT4 (ng/dL)	1.14 ± 0.19	1.05 ± 0.45	n.s	1.06 ± 0.16	n.s
Total protein (g/dL)	7.11 ± 0.57	6.63 ± 0.95	n.s	7.21 ± 0.80	n.s
Albumin (g/dL)	4.08 ± 0.47	3.60 ± 0.69	<0.05	3.81 ± 0.80	n.s
AST (U/L)	30.6 ± 27.2	38.3 ± 29.0	n.s	27.3 ± 16.0	n.s
ALT (U/L)	70.2 ± 150.3	33.1 ± 25.5	n.s	28.2 ± 20.2	n.s
Creatinine (mg/dL)	0.65 ± 0.22	2.06 ± 1.70	<0.005	1.52 ± 0.74	<0.0005
Urea nitrogen (mg/dL)	14.0 ± 6.9	63.0 ± 59.2	<0.005	47.2 ± 28.0	<0.0005
eGFR (mL/min/1.73m^2^)	102.6 ± 43.4	49.7 ± 40.3	<0.0005	43.9 ± 22.7	<0.005
Total cholesterol (mg/dL)	205.1 ± 58.2	193.3 ± 64.3	n.s	241.3 ± 46.6	n.s
Triglyceride (mg/dL)	195.4 ± 143.1	206.2 ± 64.3	n.s	230.7 ± 111.6	n.s
LDL-cholesterol (mg/dL)	127.2 ± 55.9	104.3 ± 58.3	n.s	131.0 ± 65.2	n.s
HDL-cholesterol (mg/dL)	43.0 ± 14.5	42.4 ± 19.0	n.s	55.1 ± 22.5	n.s
UA (mg/dL)	5.01 ± 2.18	13.19 ± 7.53	<0.0005	7.85 ± 2.95	<0.05
CRP (mg/dL)	2.20 ± 5.25	4.87 ± 6.60	n.s	5.49 ± 10.85	n.s

Data presented as mean ± standard deviation. BMI, body mass index; TSH, thyroid-stimulating hormone; FT3, free thyroxin 3; FT4, free thyroxine 4; LDL-cholesterol, low-density lipoprotein cholesterol; HDL-cholesterol, high-density lipoprotein cholesterol; eGFR, estimated glomerular filtration rate; UA, uric acid; CRP, C-reactive protein; n.s, not significant.

### Changes in Thyroid Hormone Levels Before and After Treatment

**Figure 1:**
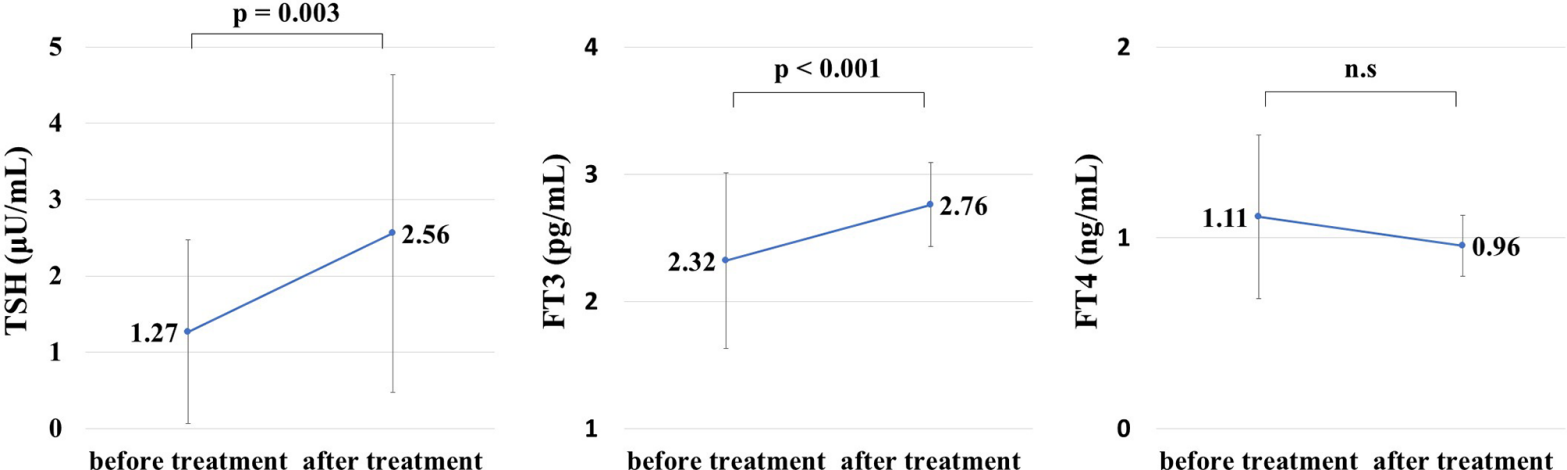
Thyroid hormone levels before and after treatment for hyperglycemia during hospitalization. Thyroid hormone levels after treatment for hyperglycemia were measured on a mean of 13.2 days after admission. Thyroid-stimulating hormone (TSH) and free thyroxine (FT3) levels were significantly improved after treatment for hyperglycemia (TSH: 1.27 ± 1.20 μU/mL before treatment, 2.56 ± 2.08 μU/mL after treatment, p = 0.0026, FT3: 2.32 ± 0.69 pg/mL before treatment, 2.76 ± 0.33 pg/mL after treatment, p = 0.00037). NS, not significant.

Thyroid hormone levels were retested on a mean of 13.2 days after initiation of treatment for hyperglycemia-related metabolic disorders. The changes in thyroid hormone levels before and after treatment are shown in **Figure 1**. TSH (μU/mL) increased from 1.27 ± 1.20 to 2.56 ± 2.08 (p = 0.0026). FT3 (pg/mL) increased from 2.32 ± 0.69 to 2.76 ± 0.33 (p = 0.00037). There was no significant change in FT4 between before and after treatment.

### Changes in Various Parameters With and Without NTI

The frequency of NTI was 80% in DKA group, 70% in HHS group and 50% in DK group. Thyroid function in patients without and with NIT is as follows: In patients without NTI, TSH 1.57 ± 0.72 μU/mL, FT3 2.29 ± 0.57 pg/mL, FT4 1.07 ± 0.37 ng/dL; in patients with NTI, TSH 0.94 ± 1.05 μU/mL, FT3 2.29 ± 0.57 pg/mL, FT4 1.14 ± 0.18ng/dL. TSH and FT3 were lower in patients with NTI compared with those without NTI (p=0.0029, p=0.0015). There was no difference in FT4. Total ketone body, 3-hydroxybutyrate, uric acid and CPR were significantly higher in patients with NTI (p=0.0052, p=0.0042, p=0.024, p=0.050) (**Table 2**).

**Table 2:** Comparison of various parameters in subjects with and without NTI.

Parameter	with NTI (n=34)	without NTI (n=18)	p value
Blood glucose levels (mg/dL)	584.0 ± 233.5	600.3 ± 274.5	n.s
HbA1c (%, NGSP)	12.3 ± 3.0	12.9 ± 3.8	n.s
Total ketone body (μmol/L)	7927.0 ± 5346.7	3655.9 ± 3840.5	<0.05
Acetoacetic acid (μmol/L)	2393.0 ± 1692.0	1240.8 ± 1390.5	<0.05
3-Hydroxybutyric acid (μmol/L)	5470.6 ± 3806.4	2415.1 ± 2415.1	<0.005
TSH (μU/mL)	0.94 ± 1.05	1.57 ± 0.72	<0.005
FT3 (pg/mL)	2.29 ± 0.57	2.95 ± 0.31	<0.005
FT4 (ng/dL)	1.07 ± 0.37	1.14 ± 0.18	n.s
UA (mg/dL)	10.2 ± 7.0	6.1 ± 2.7	<0.05
CRP (mg/dL)	5.31 ± 8.50	1.05 ± 1.36	<0.05

Data presented as mean ± standard deviation. TSH, Thyroid-stimulating hormone; FT3, free thyroxin 3; FT4, free thyroxine 4; UA, uric acid; CRP, C-reactive protein; n.s, not significant.

### Evaluation About Which Factors Independently Influence FT3 Levels

**Figure 2:**
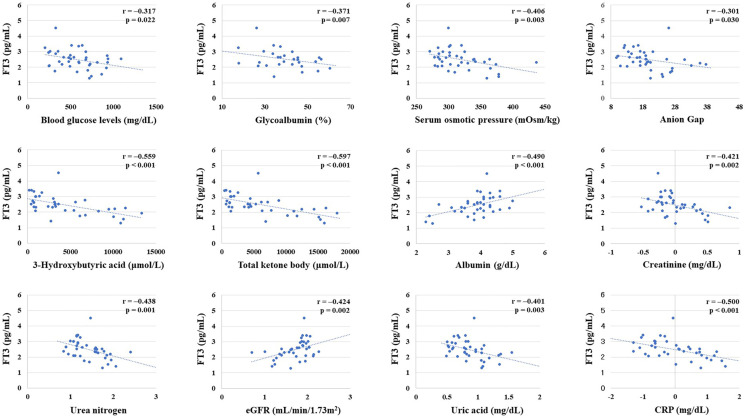
Correlation between free thyroxine 3 (FT3) level and various parameters. Creatinine, urea nitrogen, estimated glomerular filtration rate (eGFR), albumin, and CRP were converted to natural logarithm. There was a significant negative correlation between FT3 and blood glucose levels on admission, glycoalbumin, serum osmolality, Anion Gap, 3-hydroxybutyrate, total ketones, creatinine, urea nitrogen, uric acid, and CRP. There was a significant positive correlation between FT3 and albumin and eGFR.

FT3 levels were negatively correlated with blood glucose levels, glycoalbumin, serum osmolality, anion gap, 3-hydroxybutyrate, creatinine, urea nitrogen, uric acid, and CRP on admission. FT3 value was positively correlated with albumin and eGFR. Next, to examine which factors independently influence FT3 levels, we performed multivariate linear regression analysis by using variables that showed significant correlations in the above analysis as explanatory valuables and FT3 as an objective valuable. As the results, 3-hydroxybutyrate was independently associated with FT3 (**Table 3**). The correlation between these dependent variables and FT3 is shown in the scatter plot in **Figure 2** (r= −0.558703, p=0.000017, r= −0.48955802, p=0.00023).

**Table 3:** Multiple regression analysis about several factors influencing FT3 levels.

Parameter	Standard β	t value	p value
Age (years)	−0.135	−0.91	n.s
Male (%)	0.081	0.56	n.s
Blood glucose levels (mg/dL)	−0.166	−1.10	n.s
3-Hydroxybutyric acid (μmol/L)	−0.508	−3.54	<0.005
Parameter	Standard β	t value	p value
Age (years)	−0.039	−0.31	n.s
Male (%)	0.131	1.11	n.s
Blood glucose levels (mg/dL)	−0.182	−1.47	n.s
3-Hydroxybutyric acid (μmol/L)	−0.506	−4/29	<0.005
CRP (mg/dL)	−0.444	−3.78	<0.005

CRP, C-reactive protein; n.s, not significant.

## Discussion

In this study, we focused on the effect of metabolic disorders related to hyperglycemia on thyroid hormone levels in adult patients. Previous reports of pediatric cases suggested that 3-hydroxybutyrate and albumin may contribute to the development of NTI (7, 9). In adult cases, FT3 was correlated with blood glucose level, serum osmolality, ketosis, albumin, renal function, and CRP on admission. In addition, 3-hydroxybutyrate and albumin were independently associated with FT3. In contrast to previous studies in pediatric patients, no significant correlations were found between TSH, FT4, and other parameters in adult patients. In general, serum level of each thyroid hormone changes with time after the onset of NTI, and many patients with NTI are usually treated at a relatively early stage of the disease. We assume that such background can, at least in part, explain the reason why only FT3 level was associated with various parameters in this study (2).

This is a single-center, retrospective, observational study. Patients admitted for DKA, HHS, and DK with hyperglycemia and metabolic ataxia were included and analyzed. While few studies have reported that HHS causes NTI, it is very interesting that HHS may affect thyroid function in addition to ketosis. On the other hand, since the number of HHS patients hospitalized during the study period was small, further detailed analysis with a larger number of subjects would be necessary to strengthen our hypothesis obtained in this study.

Since true hypothyroidism is involved in pathophysiology such as circulatory dynamics and fluid retention, it is not uncommon to struggle with the decision of replacement therapy, especially in critically ill patients. On the other hand, it has been reported that thyroid hormone replacement therapy for NTI in patients admitted to the ICU does not show beneficial effect on subsequent outcome, but rather suppresses TSH secretion and inhibits improvement of NTI (10). This study shows that NTI associated with acute metabolic disorder can be improved by treatment for primary disease in a couple of weeks without thyroid hormone replacement therapy.

To the best of our knowledge, this is the first report showing the influence of hyperglycemia-related metabolic disturbance on thyroid function in adult subjects. The data in this study clearly shows that transient NTI is frequently observed in subjects with acute metabolic disorders such as DKA, HHS and DK. In addition, we should bear in mind that thyroid hormone replacement therapy is not necessary in subjects with NTI due to DKA, HHS and DK, especially when overt symptoms of hypothyroidism are not observed.

## Data Availability Statement

The raw data supporting the conclusions of this article will be made available by the authors, without undue reservation.

## Ethics Statement

The studies involving human participants were reviewed and approved by Institutional Review Board of Kawasaki Medical School. Written informed consent for participation was not required for this study in accordance with the national legislation and the institutional requirements.

## Author Contributions

YI researched data and wrote the manuscript. TK, FT, TS, MK, EN, KD, RW, HI, KT, JS, YF, YK, MS, SN, TM, and KK participated in discussion. HK participated in discussion and reviewed the manuscript. All authors contributed to the article and approved the submitted version.

## Conflict of Interest

HK has received honoraria for lectures, received scholarship grants, and received research grant from Novo Nordisk Pharma, Sanofi, Eli Lilly, Boehringer Ingelheim, Taisho Pharma, Sumitomo Dainippon Pharma, Takeda Pharma, Ono Pharma, Daiichi Sankyo, Mitsubishi Tanabe Pharma, Kissei Pharma, MSD, AstraZeneca, Astellas, Novartis, Kowa, Abbott. KK has been an advisor to, received honoraria for lectures from, and received scholarship grants from Novo Nordisk Pharma, Sanwa Kagaku, Takeda, Taisho Pharma, MSD, Kowa, Sumitomo Dainippon Pharma, Novartis, Mitsubishi Tanabe Pharma, AstraZeneca, Boehringer Ingelheim, Chugai, Daiichi Sankyo, Sanofi.

The remaining authors declare that the research was conducted in the absence of any commercial or financial relationships that could be construed as a potential conflict of interest.

## Publisher’s Note

All claims expressed in this article are solely those of the authors and do not necessarily represent those of their affiliated organizations, or those of the publisher, the editors and the reviewers. Any product that may be evaluated in this article, or claim that may be made by its manufacturer, is not guaranteed or endorsed by the publisher.
